# The Effect of an Intervention Based on the PRECEDE- PROCEED Model on Preventive Behaviors of Domestic Violence Among Iranian High School Girls

**DOI:** 10.5812/ircmj.3517

**Published:** 2013-01-05

**Authors:** Yalda Soleiman Ekhtiari, Davoud Shojaeizadeh, Abbas Rahimi Foroushani, Fazlollah Ghofranipour, Batoul Ahmadi

**Affiliations:** 1 Department of Health Education and Promotion, School of Public Health, Tehran University of Medical Sciences, Tehran, IR Iran; 2 Department of Epidemiology and Biostatistics, School of Public Health, Tehran University of Medical Sciences, Tehran, IR Iran; 3 Department of Health Education, Tarbiat Modares University, Tehran, IR Iran; 4 Department of Management and Health Economy, School of Public Health, Tehran University of Medical Sciences, Tehran, IR Iran

**Keywords:** Domestic Violence, Health Education, Health Promotion, Iran

## Abstract

**Background:**

Domestic violence is one of the major health problems among women. Promoting preventive behaviors of domestic violence among women and girls can play crucial role in reducing this health problem.

**Objectives:**

This study was conducted to evaluate the effect of an intervention based on PRECEDE-PROCEED Model on preventive behaviors of domestic violence among Iranian high school girls.

**Patients and Methods:**

An interventional study was completed during 2010-2011 in 10 high schools in the district 17 of Tehran municipality with 510 female students. We used the components of the PRECEDE-PROCEED Model for planning, implementation and evaluation of the program. Based on the results of need assessment, an appropriate environmental and educational intervention was implemented in the intervention group. Changes in predisposing, reinforcing, enabling factors and especially preventive behaviors immediately and two months after the intervention activities were assessed by questionnaires based on PRECEDE-PROCEED Model.

**Results:**

The intervention had significantly positive effect on predisposing, enabling and reinforcing factors immediately and two months after the intervention (P < 0.05). Repeated measures Analysis of variance showed a significant positive increase in preventive behaviors score in the intervention group from baseline to two months.

**Conclusions:**

The PRECEDE-PROCEED Model can be applied as a conceptual framework for identifying the relevant behavioral and environmental risk factors associated with domestic violence. Development and implementation the skills-based education using this model can lead to the promotion of preventive behaviors of domestic violence and reduction in domestic violence cases.

## 1. Background

Domestic violence is defined as one of the major health problems among women, which can involve any women regardless of her characteristics such as regional, social, and cultural characteristics (1). In 1997, World Health Organization (WHO) defined domestic violence as "the range of sexually, psychologically, and physically coercive acts used against adult and adolescent women by current or former male intimate partners"(2). Both short-term and long-term consequences of domestic violence can affect women's health directly or indirectly and lead to wide range of physical and mental disturbances (1). In 2005, results of a study on women's health and violence against women, which was done in 10 countries, showed that the lifetime prevalence of physical and/or sexual violence by an intimate partner varied from 15% in Japan to 71% in Ethiopia (3). Iran is no exception to the rule, where this health problem exists in Iran too (4). However, accurate statistics are not available in this area. Since some studies consider domestic violence as a continuing hidden epidemic (5), governmental and nongovernmental organizations in collaboration with international organizations need to give priority to developing and implementing the preventive programs of domestic violence (6). According to studies in Iran, introducing women and girls to related factors of domestic violence, empowering them to have appropriate reactions in their communication, reduce potential conflicts in their relationships with others, and adopt correct alternative behaviors during problem-solving processes, can be effective in preventing or reducing domestic violence cases (7). In health education field, certain models help us explain occurrence behavior and conduct health education program in order to view its effect on behavior (8). One of the frequently used models in health education and promotion is the PRECEDE-PROCEED Model. According to most recent version of the model by Green and Kreuter (2005), this model prescribes eight phases in planning, implementing, and evaluating health promotion programs. The PRECEDE portion of the model (Phases 1-4) includes social, epidemiological, behavioral, environmental, educational, administrative, and policy assessments. The PROCEED portion of the model (Phases 5-8) includes implementation, process evaluation, impact evaluation, and outcome evaluation. The first portion of the model focuses on program planning and the second portion focuses on implementation and evaluation (9). Schools have been identified as a key setting for primary prevention activities and promotion of the preventive behaviors of domestic violence among youth (3). Hence, we studied the effect of an intervention based on PRECEDE PROCEED Model on promoting preventive behaviors of domestic violence among Iranian high school girls.

## 2. Objectives

This study was conducted to evaluate the effect of an intervention based on PRECEDE-PROCEED Model on preventive behaviors of domestic violence among Iranian high school girls.

## 3. Patients and Methods

This study was interventional, and was conducted during 2010-2011 on 510 12'th grade senior third grade high school girls in district 17 of Tehran municipality. This district has characteristics such as high density populated community and low level of socioeconomic situation. The sample size was calculated with an 80% confidence interval and 5% accuracy. Considering a design effect on sample size, it was determined that the sample size in each of the interventions and the control group to be 255. Cluster sampling method was used. All high schools in this district were selected and each school was identified as a cluster. Schools were divided into two groups, each group included five schools. Then from 12'th grades senior students of each school, a sample size was randomly selected. Study inclusion criteria included being enrolled in the 12'th grade. Informed consent was obtained from each participant included in the study. The study protocol conforms to the ethical guidelines of the 1975 Declaration of Helsinki. At first, through a mixed qualitative and quantitative study, the first four steps' assessment of PRECEDE portion were conducted and then an appropriate intervention based on their results was developed, implemented, and evaluated. Qualitative data were collected through four focus group discussions with the participation of high schools girls, and from five in-depth interviews with key informants related to domestic violence, such as women's health specialists and consultants. Quantitative data were collected through the questionnaire developed by the researcher, that was based on PRECEDE-PROCEED Model and included demographic characteristics, predisposing factors that included knowledge (7 questions) and attitude (26 questions based on Likert scale), enabling factors (4 questions), reinforcing factors (4 questions), and behavior (9 questions) sections. Scores of variables were classified as weak (less than 50 percent), moderate (50-70 percent), and good (> 70 percent) level. Ten health education and health promotion professionals confirmed the face and content validity of the questionnaire. The reliability of the attitude questionnaire was confirmed with a Cronbach alpha reliability coefficient of 0.71, obtained in a pilot study on 30 students other than the two main study groups. Test- Retest method used for determining the reliability of the knowledge, reinforcing, and, enabling questionnaires in the pilot sample, has been achieved with a correlation coefficient of 0.75, 0.80, and 0.77, respectively.

### 3.1. Ethical Consideration

The study was approved by Tehran University of Medical Sciences. All Ethical issues - informed consent, conflict of interest, plagiarism, misconduct, data fabrication and/or falsification, double publication and/or submission, redundancy, etc.- have been considered carefully by the authors. The respondents were anonymous and participated willingly and voluntarily in this study

### 3.2. Social Assessment

In this phase, the researchers identified factors affecting health outcomes and quality of life in the target population. We used some methods for data collection such as interviews with key informants and focus group discussions with high school girls. Results showed that domestic violence can be one of the social problems in our country, which can affect girls' and women's health, and respondents partly confirmed the existence of this problem in our society.

### 3.3. Epidemiological, Behavioral, and Environmental Assessment

In this phase, we collected existing data related to domestic violence such as types, prevalence rates, importance, and factors associated with domestic violence in Iran and other countries, using data sources such as various online databases and national health surveys in other countries. Then, in behavioral and environmental assessments, factors causally associated with domestic violence were systematically identified, and the most important and changeable behavioral and environmental factors associated with domestic violence were found. Finally, behavioral objectives and environmental objectives were constructed for each risk factor. Results of the focus group discussions and interviews were widely applied for this step. Review of the literature and existing data on domestic violence showed that women and girls are a high-risk group for mortality and morbidity related to domestic violence, and the most important factor found in Iranian families is the lack of the preventive behaviors of domestic violence. After identifying and rating the behavioral and environmental determinants in terms of importance and changeability, the determinants were selected. According to the results of qualitative study, in terms of behavioral determinants of the performance of preventive domestic violence behaviors, life skills applications related to prevention of domestic violence were considered as the target behavior. In terms of environmental determinants of the performance of preventive domestic violence behaviors, access to places, people, or informational resources, such as educational material, classes, and electronic databases were considered as the target behavior. Some demographic variables such as parents' occupation and education, family size, birth order, housing, having a specific room, family status, and field of education were also considered as non-health factors related to quality of life in target population.

### 3.4. Educational and Ecological Assessments

This phase entails identifying the predisposing, enabling, and reinforcing factors, which leads to behavioral change. Predisposing factors are antecedents to behaviors that motivate particular health related attributes, such as knowledge, attitudes, and beliefs related to domestic violence. Reinforcing factors are provided in reward and incentive for the persistence of the health related behavior, such as getting influence from significant people. In this study, getting influences from parents especially mothers, teachers, school counselors, and peers are considered as the reinforcing factors. Enabling factors are those that facilitate performance of the health action, such as resources, skills, and supportive policies that are essential to conduct the behaviors (9). In this study, the enabling factors were availability and accessibility to counseling centers, educational classes, and informational resources, such as books, website, etc. We used the questionnaire based on the PRECEDE-PROCEED Model and the results of qualitative study to determine following factors. Finally, an appropriate educational and environmental intervention to promote preventive behaviors of domestic violence was designed based on the results of this phase.

### 3.5. Administrative and Policy Assessments

The fourth phase of the model focuses on identifying resources, policies, supports, and facilities needed for implementing and evaluating the health education program (10). We assessed a place and timetable for activities, budgeting, personnel, organizational barriers, facilitators, policies, responsibilities, necessary supports and coordinations for implementing educational and environmental interventions. These items were mainly identified through the interviews with key informants. After these quadric assessments, the program's components were determined. Educational objectives, content of the educational program, messages, concepts, and materials were developed through finding expert's views and reviewing the scientific resources. Now, it was time to implement the program in the intervention group. The environmental intervention plan was also developed.

### 3.6. Implementation

After planning the intervention, the proposed program was implemented among students of the intervention group as following: 1. To increase students' awareness about domestic violence prevention: holding lectures for life skills education and colloquy sessions about domestic violence prevention twice a week until reaching the educational objectives, distribution of the educational pamphlets related to domestic violence prevention to students, and creating an educational web log about life skills related to domestic violence prevention. 2. To change students' attitude: holding focus group discussions with high school girls about issues related to domestic violence, and benefits and barriers of domestic violence prevention, until reaching the correct attitudes towards domestic violence prevention. 3. To change student's behavior: role-playing with high school girls to improve skills associated with domestic violence prevention. 4. To promote the reinforcing factors: advocating and training high schools counselors to effectively conduct domestic violence prevention education for the students, distribution of the educational booklets to parents to involve them, especially mothers in the violence prevention education to their daughters, and reinforce messages learned at high school. 5. To promote the enabling factors: coordination with available and free counseling centers in selected district and introducing them to high school girls, introducing teachers and high schools counselors as enabling factors to students, and provision of the correct information to increase their awareness about domestic violence prevention through the introduction of books and reliable websites related to domestic violence prevention.

### 3.7. Process Evaluation

Process evaluation occurs during implementation of the program and is used to evaluate the process by which the program is being. In this phase, achieving the educational objectives is measured (11). In this study process evaluation includes evaluating the program components such as the program staff, methods, materials used, and activities.

### 3.8. Impact Evaluation

This phase determines the immediate effect of the program on the target behavior, and it occurs after the program ends (9). In this study, impact evaluation consists of assessing changes in predisposing, reinforcing, enabling, and behavioral factors immediately after and two months after intervention activities through analysis of the questionnaires.

### 3.9. Statistical Analysis

The data was analyzed using the SPSS 18 statistical software, using chi-square and analysis of variance with repeated measures tests. Baseline demographic characteristics before the intervention in both groups were compared using the Chi-square test for categorical variables. To determine the effects of the intervention, repeated measures analyses of variance were done. Time was the within-subject factor with three levels (pre-test, post-test, and post-post-test). Group was the between-subject variable with two levels (the intervention and control group). When an interaction effect between group and time was found, the mean between group difference and the 95% confidence interval (CI) were reported. To further analyze the responses of the groups, the mean within group difference and the 95% (CI) were calculated for all confounding factors. For each confounding factor, a repeated measures analysis of variance was conducted separately. The goal of this phase is determining the long-term effect of the program (9). Due to time restriction on access to students and the coincidence with the summer holidays, evaluation of the effect of the intervention activities on indicators of quality of life and health status in log-term has not been evaluated yet. This program is still being evaluated and now only the short-term outcomes have been evaluated.

## 4. Results

**Figure 1:**
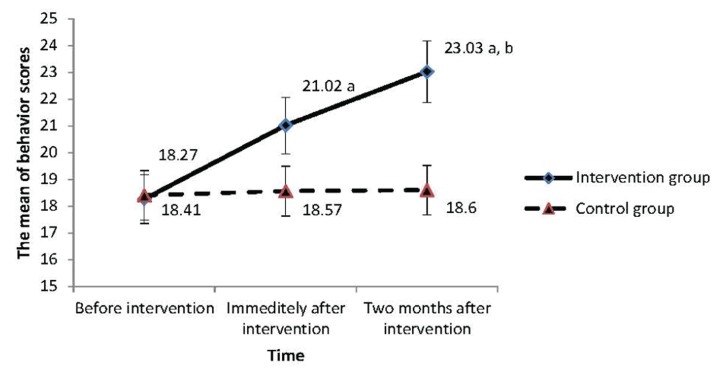
The mean of behavior score divided into the intervention and control group over time Result of a repeated measures analysis of variance: a (P < 0.001) compared to the control group, b (P < 0.001) significant difference in change scores over time

Demographic characteristics at baseline are summarized in Table 1. No significant differences were found between the intervention and the control group in terms of the demographic measures at baseline. Before intervention, there was no significant difference between two groups regarding the mean of knowledge, attitude, enabling, reinforcing factors, and behavior scores. Before intervention, the mean of knowledge score was in the weak level in the two groups, but immediately after and two months after intervention it reached to the good level in the intervention group, but no change was found in the control group. In terms of the mean of attitude score, it had no differences over time in the control group, but it reached from the moderate level to good level immediately after and two months after intervention in the intervention group. The mean of enabling and reinforcing factors scores were also in weak level in both groups at baseline, but immediately after and two months after intervention they reached moderate level in the intervention group, and in the control group they remained in the weak level. In this study, promotion of the preventive behaviors of domestic violence was the main expected objective. , The means and standard deviations for the preventive behaviors were calculated at baseline, immediately after intervention, and two months after intervention in both the intervention and the control groups where the results are shown in Table 2. The findings showed that the mean of the behavior score changed from 18.27 (weak level) in baseline to 21.02 (weak level) immediately after intervention, and finally to 23.03 (moderate level) two months after intervention in the intervention group. But in the control group, there was no significant change in the mean of behavior score over time. As shown in Table 3, the mean of behavior score over time in students of the intervention group was higher than the control group (P < 0.001). In addition, the mean of preventive behaviors score immediately after and two month after the intervention was higher in the intervention group (P < 0.001). There was a significant correlation (P < 0.001) between the type of group and the mean of preventive behaviors score. In other words, changes in the mean of preventive behaviors scores depend on which group the student belongs to. In fact, the intervention implemented was effective and the effect of the intervention was different in different times. Figure 1 confirms these results. The trend of changes in the behavior score over time was significant, so that this trend was very flat in the control group but had abrupt increase in the intervention group. Then significant effect of the intervention on the mean of preventive behaviors score for confounding factors was adjusted. As it can be found in Table 4, after the adjustment for each confounding factor, the intervention had still a significant positive effect on the mean of preventive behaviors score (P < 0.001).

**Table 1:** Demographic Characteristics of Students in the Intervention and the Control Group

Characteristics	The intervention group, No. = 255	The control group, No. = 255	P value
**Field of education, %**			0.168
Mathematics	27.1	29	
Experimental sciences	40.4	32.5	
Human sciences	32.5	38.4	
**Family size, %**			0.148
3	7.8	6.3	
4	40	36.9	
5	36.1	35.3	
6 and higher	16.1	21.6	
**Birth order, %**			0.119
First	51.4	42.7	
Second	22	27.8	
Third	16.1	14.5	
Forth and higher	10.6	14.9	
**Father's occupation, %**			0.840
Employee	33.7	29.8	
Worker	20	20	
Self employed	37.3	42	
Non employed	4.3	3.9	
Divorced or widowed	4.7	4.3	
**Mother's occupation, % **			0.537
Housewife	88.9	87.1	
Employed	9.4	11.1	
**Father's Education, %**			
Illiterate	4.3	8.6	
Less than High school	41.2	40.4	
High school Diploma	40	35.3	
Higher education	9.8	11.4	
Divorced or widowed	4.7	4.3	0.311
**Mother's education, % **			0.461
Illiterate	5.5	8.6	
(Less than) High school	54.5	52.2	
High school Diploma	32.2	30.6	
Higher education	6.7	8.6	
**Housing, %**			0.637
Rented	33.7	31.8	
Owned	66.3	68.2	
**Having a specific room, %**			0.329
Yes	50.6	54.9	
No	49.4	45.1	
**Family status, %**			0.697
Two-parent family	94.9	94.1	
Single-parent family	5.1	5.9	

**Table 2:** Mean and Standard Deviation of Behavior Score Variable in the Intervention and the Control Group at Baseline, Immediately After Intervention, and Two Months after Intervention

Groups	Before Intervention, Mean ± SD	Immediately After Intervention, Mean ± SD	Two Months After Intervention, Mean ± SD
**Intervention group**	18.27 ± 2.97	21.02 a ± 3.31	23.03 b ± 3.77
**Control group**	18.41 ± 2.95	18.57 ± 2.83	18.60 ± 2.86

^a^P < 0.001, compared to the control group

^b^P < 0.001, Significant difference in change of scores over time for intervention group.

**Table 3:** Results of Analysis of Variance with Repeated Measures for Behavior Score Related to the Intervention and the Control Group

Sources	Sum of Squares	Df a	F	P value
** Time**	1577.813	1.701	216.590	< 0.001
** Group**	1938.094	1	87.029	< 0.001
** Time group**	1340.851	1.701	184.062	< 0.001
** Error**	3700.669	864.274	-	-

^a^Abbreviation: Df: degree of freedom

**Table 4:** Results of Analysis of Variance with Repeated Measures for Behavior Score After Adjustment for each Confounding Factor

Model a	Sum of squares for group	df	F	*P *value
** Model 1**	1873.796	1	84.046	< 0.001
** Model 2**	1050.742	1	47.16	< 0.001
** Model 3**	1294.633	1	57.926	< 0.001
** Model 4**	915.125	1	41.351	< 0.001
** Model 5**	477.259	1	21.889	< 0.001
** Model 6**	452.311	1	20.499	< 0.001
** Model 7**	485.798	1	31.433	< 0.001
** Model 8**	1736.534	1	77.712	< 0.001
** Model 9**	1915.224	1	87.518	< 0.001
** Model 10**	409.470	1	18.327	< 0.001

^a^Model 1, set for field of education, Model 2, set for family size, Model 3, set for birth order, Model 4, set for father's occupation, Model 5, set for mother's occupation, Model 6, set for father's education, Model 7, set for mother's education, Model 8, set for housing, Model 9, set for having a specific room, Model 10, set for family status

## 5. Discussion

It is worth mentioning that this is the first study to apply the PRECEDE-PROCEED Model promoting preventive behaviors of domestic violence with young girls. During the last few decades, health education models were applied to achieve large-scale behavioral changes, (12) and PRECEDE-PROCEED Model has been applied in planning and implementation most of health promotion programs. Green and Kreuter proposed the PRECEDE-PROCEED Model as a conceptual framework for identifying multiple factors that affect health behaviors, health status, and the quality of life (11). Result of studies showed that it could apply in a wide variety of settings such as schools and others (13, 14). The purpose of this study was to consider the impact of an intervention based on PRECEDE PROCEED Model on preventive behaviors of domestic violence among Iranian high school girls. Our findings indicated that PRECEDE-PROCEED Model is an appropriate model for planning and implementing preventive intervention for domestic violence. In this study, results of the impact evaluation revealed significantly positive knowledge and attitude changes in the intervention group students over time. Also, reinforcing and enabling factors showed significantly positive changes immediately after and two months after intervention in comparison with the control group where there were no changes. The focus of the intervention was on promoting preventive behaviors of domestic violence. Before implementation of the intervention, both qualitative and quantitative studies consistently confirmed lack of necessary behavioral skills for prevention of domestic violence among high school girls. However, a significant increase in adoption of preventive behaviors of domestic violence immediately and two months after the intervention was observed in the intervention group students. Results of this study and other studies indicated that Skills-based education is an effective way to enable students to reduce violence and promote non-violent behaviors (6, 15). One possible explanation for the intervention's success in promoting preventive behaviors of domestic violence is that at first, we provided the rationale or motivation for target behaviors through promoting predisposing factors, and then through promoting enabling and reinforcing factors, and finally facilitated the practice of the preventive behaviors by target population. These results are in concordance with numerous other studies, which have been carried out in different societies with different health education methods. In some of these studies, they reached to similar results of present study, using repeated measures analysis of variance, where researchers found that women in the intervention group significantly reported more adopted preventive behaviors than women in the control group after three months and six months of the intervention (16, 17). In other studies, compared to baseline, women in the intervention group adopted significantly more preventive behaviors after twelve to twenty-four months (18-20). In terms of preventive behaviors of sexual violence, women in the intervention group were more likely to use an alternative strategy and have a safer sex discussion with their partners (21). In another studies, significant increase in knowledge levels, positive attitude, and behavioral intention changes were found in experimental groups at post-test in comparison the control groups (22, 23). Recently, many studies have been conducted aimed at determining the impact of life skills programs on violence prevention in developed countries, and the results of research revealed that these programs could be effective in low and middle-income countries (24-27). In spite of the intervention's positive results on high school girls, this study had several limitations that deserve further discussions. This study lacked a follow-up to determine the long-term effects of the program on the quality of life and health status of target population in long term. Further research needs to be done to examine the long-term effects of the program on health and quality of life indicators among high school girls. In addition, our study was based on self-reported information, which could be biased by the participants. In conclusion, as this study revealed, an intervention protocol for high school girls that included specific behavioral and environmental interventions on preventive behaviors of domestic violence resulted in a significant increase in the adoption of these behaviors and finally could reduce the number of domestic violence cases among women and girls in future. We believe that the effectiveness of this intervention can be attributed to the use of the PRECEDE-PROCEED Model as a conceptual framework, which can play an important role in enhancing the quality of the planning of domestic violence prevention programs. In addition, we can implement and evaluate the health education programs through defined stages of this model. Further research is needed to examine the effectiveness of such behavioral and environmental interventions in a variety of settings including clinical settings, universities, and work places, and within different professions and sectors. Empowering women and girls to adopt preventive behaviors of domestic violence can lead to decreasing the domestic violence against women cases in our country.
